# Supplementary material for: Learning curve and surgical outcome of robotic assisted colorectal surgery with ERAS program
Source: Sci Rep. 2022 Nov 29;12:20566. doi: 10.1038/s41598-022-24665-w (PMC9709162; doi:10.1038/s41598-022-24665-w)
Supplement: Supplementary file 2 — Supplementary Information 2. [file 41598_2022_24665_MOESM2_ESM.zip › RAL Raw data-Ñ[▒K/106/OP_ñw╝╨░O▒Kñσ.pdf]

正式摘要(電子簽章：000000224353094)

W52 / W52

臺中榮民總醫院

Operation Note

[redacted]

- 手術日期：2021/08/20
- 手術醫師：VS 蔣鋒帆，CR 陳映菁，R 陳瑲夫
- 執行麻醉：VS 劉恆豪
- Anesthesia : GE
- Position : Supine
- Aseptic agents : Soap , Betadine Alcohol , Betadine Aqua

Diagnosis

- Pre-operative diagnosis : Malignant tumor of cecum with obstruction and liver metastasis
- Post-operative diagnosis : Malignant tumor of cecum with obstruction and liver metastasis, s/p Robot assisted right hemicolectomy on 20210819

Surgical Indication

- tumor resection and pathologic proof

Operation

- 1. Robotic (XI) assisted right hemicolectomy, with side-to-side anastomosis, GIA 80/3.8*2, re-inforcement with silk (+)
  2. Omentectomy (+)
  3. Ileo-colic artery, right colic artery and right branch of middle colic vessels, were both ligated.
  4. Take down from the hepatic flexure : (+)
  5. Cut margin: proximal (ileum) 15 cm, distal 11 cm.
  6. Repair of mesentery defect: nil
  7. Drainage tube was inserted to Morrison pouch.
  8. Adhesion barrier film was placed below the fascia of the para-umbilical wound

Finding

- 1. A 7*5cm Borrmann type II annular tumor at cecum, which invaded to serosa layer, with partial obstruction, liver and omentum metastasis. Multiple LNs. T4N2M1c.
  2. Sever liver metastasis

Procedure

- General endotracheal anesthesia was induced and then the patient was placed in supine position. Preoperative antibiotics were given. A Foley catheter and nasogastric tube were placed. The abdomen was prepped and draped in standard sterile fashion.
  A paraumbilical vertical incision was made and a 8 mm trocar was inserted into the peritoneal cavity by open method. Peumoperitoneum was established with CO2 inflation. Davinci camera was inserted and then other three 8mm trocars were inserted over LUQ, LLQ of abdomen. Another 12mm trocars was inserted over LUQ (A) area. The operative findings were mentioned as above. The small bowel was inspected and retracted to downward by grasper. The peritoneum overlying the mesentery was then scored with electrocautery and the ileocolic artery and the adjacent vein were identified, ligated with endoclips, and transected via the electrical cautery instruement of XI system. Omentum over the proximal T-colon was freed from the transverse colon and lesser sac was entered. The proximal T-colon was dissected carefully until it was fully mobile. After the vessel were all under controlled, the colon was freed from its peritoneal attachments along the line of Toldt from the cecum to the hepatic flexure. Hemostasis was checked in the operative field. Cecum, ascending colon and hepatic flexure were all freely mobilized. Omentumectomy was done. We extended para-

- umbilical incision and then right side colon was pulled out from the wound, after transection of mesentary, side-to-side anastomosis was made by GIA-80-3.8*2. Then re-enforced by 3-0 mersilk. 12mm balloon trocar was inserted through para-umbilical wound and then pneumoperitoneum was built again. Laparoscopic camera was inserted. The possible bleeder was checked thoroughly. Adhesion barrier was placed under the extended wound.
  The extended fascia was closed with figure of 8 sutures via No.1 Vicryl. The fascia of the remaining trochars were all closed with interrupted method via 1-0 Vicryl. Massive N/S was applied for wound irrigation. The skin wound was approximated with tissue glue. The patient tolerated the procedure well and was brought to the POR in stable condition.
- Estimated blood loss: minimum
- 器械數量: 正確
- 紗布數量: 正確

Image

- PAINT
  - colon & rectum

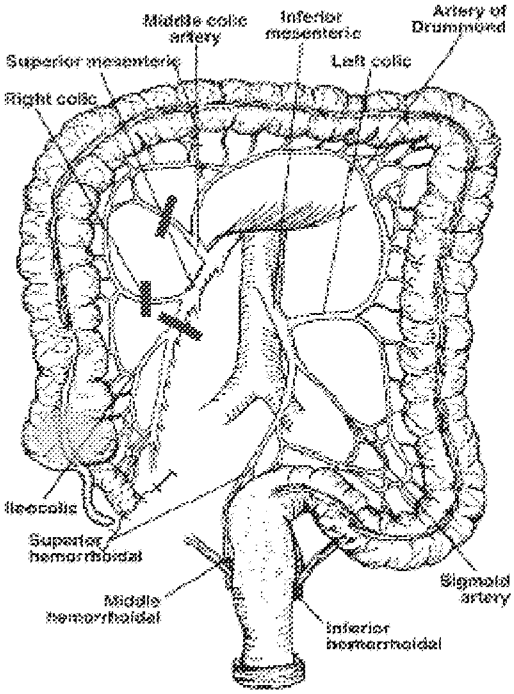

  - abdomen

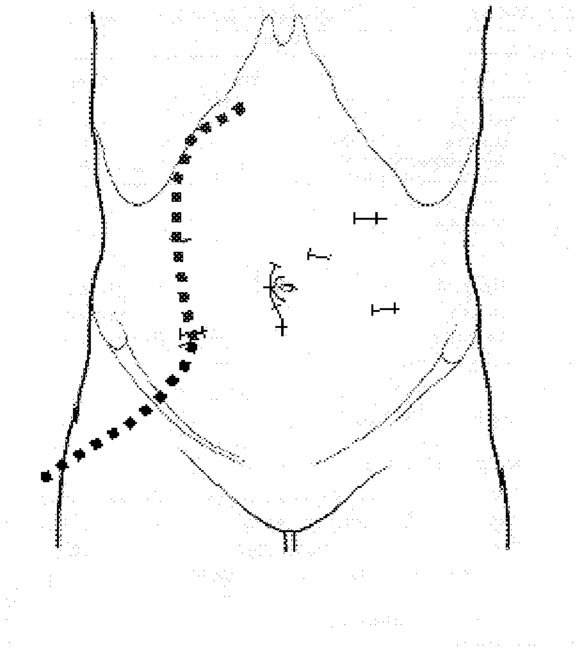

主任 2062F蔣鋒帆 / 醫師 2303K陳映菁

正式摘要(電子簽章：000000224353094)

W52 / W52

doctorRank:B
